# Religious and cultural influences on domestic violence attitudes and responses in UK ethnic minority and migrant communities: A qualitative inquiry

**DOI:** 10.1177/26330024251391813

**Published:** 2025-11-03

**Authors:** Romina Istratii, Natalia Paszkiewicz, Aysha Ahmed, Elsabeth Gezahegn King, Parveen Ali, Gene Feder, Mayra Teck Ascurra

**Affiliations:** Institute of Domestic Violence, Religion & Migration Courtenay House, Exeter, England; 4913 SOAS University of London, UK; 5195 University of Bedfordshire, UK; Ihit Le Ihitoch Women’s Support Group, UK; 7315 The University of Sheffield, UK; and Doncaster and Bassetlaw Teaching Hospitals, UK; 152331 Bristol Medical School, UK; Institute of Domestic Violence, Religion & Migration, UK

**Keywords:** Community resources, domestic violence and abuse (DVA), ethnic minorities, faith communities, migration, religious mediators, services, United Kingdom

## Abstract

The current paper presents findings from qualitative research on domestic violence and abuse (DVA) with Christians and Muslims from Ethiopian, Eritrean, and Bangladeshi communities in the United Kingdom (UK). The study explored understandings of and attitudes toward DVA and responses that integrated cultural and religious institutions and resources. Qualitative research was conducted with the help of community-based researchers with existing links and relationships of trust in the respective communities. Three researchers conducted 16 interviews and one focus group discussion with eight participants (total *n* = 24). The study added to the existing evidence on barriers that DVA victims from ethnic minority faith communities face in migration contexts, adding insight into the complex interactions between norms and pressures at country of origin and conditions in the host society, and the role of religious beliefs as culturally contextualized in this relationship. The study also reinforced the significant role that religious establishments and mediators can play in domestic violence responses, but also their general unpreparedness to do so. Participants proposed that integrating religious institutions and resources in DVA responses would be an effective way forward.

## Introduction

Whilst migrant communities are not reported to experience domestic violence and abuse (DVA) more frequently than non-migrant communities, they face migration-specific vulnerabilities that can contribute to DVA and create additional barriers to accessing support (Du Monte and Forte, 2012; Holtmann and Rickards, 2018; Istratii, 2023). In the United Kingdom (UK), such vulnerabilities are also shaped by what has been termed the “hostile environment,” a policy framework introduced through the Immigration Acts of 2014 and 2016 that restricts migrants’ access to public services, housing, and welfare support. This has had significant implications for migrant women, as an insecure immigration status can heighten dependency on abusive partners, deter help-seeking, and limit access to protection or specialist services (Istratii, 2023; Khelaifat, 2018). The UK’s hostile policy environment has been associated with women being excluded from accessing domestic violence services due to their immigration status and losing their right to remain in the UK if their relationship ends during their first 5 years of residing in the country (Sedacca, 2024: 75).

Khelaifat’s (2018) systematic review of qualitative studies globally and primary research in the UK reported constrained help-seeking and receiving experiences of migrant women affected by DVA due to a lack of access and knowledge, immigration status, and language barriers, as well as a fear of breach of confidentiality. A study with Eritrean asylum-seekers in Israel identified reasons including a victim’s legal situation in the host country, co-dependence on a husband’s immigration status, economic precarity, and a lack of institutional support to exit abusive relationships (Gebreyesus et al., 2018). Similarly, research into perspectives on DVA among Ethiopian women in Australia reported threats of deportation and deliberate social isolation used by abusive partners to silence and control women (lemma et al., 2023: 719). Other causes may include the continuation and prevalence of patriarchal norms associated with societies of origin or racist attitudes in host societies toward ethnic minorities (Du Monte and Forte, 2012; Holtmann, 2016; Holtmann and Rickards, 2018). In many communities, DVA may also not be discussed due to socially ascribed norms reinforcing stigma and shame, coupled with a fear that the community might be labeled as inherently problematic or violent (Du Mont and Forte, 2012; Holtmann, 2016).

Consequently, most cases of DVA in migrant communities are not formally reported (Holtmann and Rickards, 2018; Pan et al., 2006). A previous study engaging the Bangladeshi community in the United States (US), for example, demonstrated a lack of acceptance that abuse happens, an attitude associated with an increased number in women’s deaths (Bhandari, 2022: 73). In such conditions, DVA victims/survivors^1^ may turn inwards to manage their situations, including seeking mediation through cultural and religious institutions and practices (e.g., Al-Natour et al., 2019; Holtmann and Rickards, 2018; Istratii, 2020). However, cultural and religious expectations around marriage and the conjugal relationship, coupled with gender unequal social norms, can contribute to victims staying in abusive relationships (Istratii, 2020; Le Roux and Pertek, 2023). A study about experiences of Eritrean refugee women in the UK found that religious institutions advised women to tolerate abusive partners (Tsegay and Tecleberhan, 2023: 11). Often DVA victims will try to endure abusive situations by praying and reading sacred texts as a source of strength and comfort (Aghtaie et al., 2020; Al-Natour et al., 2019; Gillum et al., 2006; Raday, 2012).

A main challenge in overcoming attitudes that maintain women’s abuse is a tendency in faith communities to frame cultural norms in religious discourse, making social and normative change more difficult. In her study of spousal abuse in the Ethiopian Jewish (Beta Israel) community in Israel, Kacen revealed a faith-based worldview in the community that resulted in a discourse on violence whereby what was deemed cultural was closely interwoven with faith parameters, making it nearly impossible to separate the two (Kacen, 2006: 1281). Kacen argued that social and cultural expectations were often misrepresented as religious obligations, making it difficult for both community members and outsiders to distinguish and thus separate cultural practices from religious teachings. There is sufficient anthropological and qualitative evidence to show that the effects of religious beliefs and faith on DVA responses, mediated by its culture-specific embodiments, are not straightforward and can be positive, negative, or neutral depending on the circumstances (Istratii, 2020; Istratii and Ali, 2023; Le Roux and Pertek, 2023; Nason-Clark et al., 2018). Our own long-term anthropological research on domestic violence in the Ethiopian Orthodox community in Ethiopia evidenced this with unprecedented ethnographic insight, with the author proposing a novel schematic to understand the relationship between “culture” and “religion” and how this relationship influenced the community’s willingness to be critical and revisit pernicious social norms contributing to the problem (Istratii, 2020). In essence, attitudes in the community were diverse, with some members identifying “culture” with “religion” and being unwilling to criticize cultural practices because they saw this as a deviation from religious tradition. Others saw “culture” as different from “religion” and were more concerned about the problem of acculturation or modernization and the deviation from religious tradition that either of these phenomena implied. The more willing the participants were to differentiate the two, the more open they were to reconsider social norms that were harmful and that had been presented historically as “religious” (ibid.).

The role of religious institutions and mediators is documented to be equally nuanced and multidirectional. In faith communities, religious teachers and mediators tend to influence family situations through mediation and they are often the first point of reference when domestic violence occurs, especially in rural communities or ethnic minority contexts where access to other services may be limited (Istratii, 2020; Le Roux and Bowers Du Toit, 2017; Le Roux and Loots, 2017). However, the international evidence show that clerics and spiritual advisors are usually not prepared to respond to DVA with awareness of safeguarding, trauma-sensitivity and the theological confidence required to challenge deeply embedded social norms (Istratii and Ali, 2023; Istratii and Kalkum, 2023). As a result, their responses on the ground can vary, with some being more unhelpful than others. Oftentimes, religious institutions and mediators perpetuate, rather than subvert, deeply patriarchal, hierarchical or gender rigid regimes, reflecting wider societal conditions (ibid.).

Our review of organized DVA services in multicultural societies, including the UK, showed an increased recognition among providers of the effect that clients’ religious and cultural backgrounds had on their responses, but found no systematic discussion of how faith-informed and culturally adapted services should be developed (Istratii et al., 2024). Some studies suggest that generalist DVA services could incorporate culturally specific elements in their recruitment strategies and intervention approaches to become more culturally resonant (see literature review in Istratii et al., 2024). One particular study from the UK found the need for both culturally specific and generalist services to be combined for more effectiveness (Burman et al., 2004). Recent research has highlighted the limitations of addressing cultural diversity without accounting for faith and religious practice. Chowdhury and Winder (2022) have also argued that DVA services must adopt an intersectional lens that acknowledges the interconnectedness of cultural, religious, migration, and gender factors in shaping both vulnerabilities and pathways to support, in order to ensure meaningful engagement with individuals and communities. Yet, these conversations have tended to focus on cultural diversity and less so on faith sensitivity and religious beliefs. The question of how faith-informed resources and religious mediators could be integrated in generic or generalist DVA services remains largely unexplored given the lack of specialized theological knowledge among providers and the different degrees of awareness and engagement among religious teachers and mediators in organized DVA responses.

Against this backdrop, the current study sought to explore the following questions:

(1) How do members of migrant and ethnic minority faith communities in the UK understand DVA and to what extent do they consider this to be a problem in their communities?
(2) What kind of responses have they identified in their communities and what is the effectiveness of such responses?
(3) How do they perceive the role of religious beliefs, institutions and mediators in responding to DVA victims/survivors and how can such responses be improved?

The study was guided by the UK’s broader policy climate, where hostile environment measures have been shown to intensify migrant women’s vulnerabilities, considerations around gender regimes and norms in the communities of study, and a recognition of the intricate and multidimensional effects of religious beliefs, institutions, and mediators on DVA responses.

## Methods

In developing a research methodology, we started from the understanding that research with minoritized communities can present challenges for open discussion due to the cumulative effects of social hierarchies, gender norms, migration experience and power differences between researchers and participants (Krueger and Casey, 2015). Our qualitative methodology was informed by a decolonial ethos seeking to place interpretative power in the hands of communities involved in the research and emphasizing confidentiality, equity, and reflexivity. We recognized that it would not be possible to conduct representative research inclusive of all ethnic minorities in the UK, so we focused on an in-depth study with a few communities where the research team had cultural, religious, and linguistic expertise, as such findings were largely missing from the literature we reviewed.

Throughout the research, the participants’ faith background was documented and considered to explore how faith intersected with cultural norms in shaping everyday life and DVA understandings. In engaging with the three communities, Ethiopian, Eritrean, and Bangladeshi, we sought to include a diversity of religious affiliations in these communities and not merely those representing the majority faith in each. Hence, we engaged both Muslim and Orthodox Christian Eritreans, Orthodox Christian and Jehovah’s Witness, Christian Ethiopians, and Muslim Bangladeshi participants. Although many participants shared a faith or denomination, ethnic and cultural context influenced markedly how faith was practiced and understood.

In terms of research methods, the researchers conducted semi-structured interviews with females and males, although one researcher carried out a female-only focus group across multiple sessions for practical reasons. Focus group discussions (FGDs) were chosen to encourage participants to share experiences collectively and to enrich qualitative insights. Recognizing that conducting FGDs with minoritized communities entails challenges related to hierarchy, gender, and power (Krueger and Casey, 2015), the study mitigated these by engaging community-based researchers with shared linguistic, cultural, or religious backgrounds, or with pre-existing trust within the community. In their engagement with participants, the researchers emphasized confidentiality, equitable participation and reflective discussion to mitigate potential barriers.

The researchers used their connections to recruit participants (e.g., through a domestic violence service provider, a community-based organization or personal relations) and leveraged their bilingual and/or bicultural knowledge and identities to build trust and foster participant openness to the research. The three researchers were Natalia Paszkiewicz (NP, Eritrean community), Aysha Ahmed (AA, Bangladeshi community), and Elsabeth Gezahegn King (EGK, Ethiopian community). The research was coordinated by lead researcher, Romina Istratii (RI) and was advised by Parveen Ali (PA), and Gene Feder (GF). RI worked with the researchers and the co-investigators to co-develop a robust research protocol and process, align the data collection and data analysis process, and co-author the final report.

Each researcher analyzed their findings through their own subjective lens, being guided by thematic codes agreed prior to the interviews deductively (following the research questions), as well as additional thematic codes that emerged inductively from their distinct research experience. Several thematic codes were similar, and a decision was made to collapse them into a single code. A few proposed thematic codes were too specific and were not used, but the distinct insights flagged by the researchers were nonetheless incorporated into the closest theme.

### Issues of positionality

Two of the researchers, AA and EGK, were recruited directly from the communities of interest and are of Bangladeshi and Ethiopian origin. Whilst NP is not of Eritrean heritage, she has had close relations with the Eritrean community through previous professional work. The main strength of such an approach was the researchers’ pre-existing trust with the communities, which fostered greater openness from the participants given the sensitivity of the topic and political or religious forces that created division or mistrust in some of these communities. Additionally, the researchers’ contextual understanding of cultural standards, religious traditions, and gender norms was crucial for interpreting these realities. The knowledge of local languages was another strength, giving participants the option to speak in their primary languages, also known by two researchers.

Whilst it was important for the researchers to be mindful of the participants’ religious affiliations and behave appropriately to that context, sharing a faith or being religious was not a criterion for recruiting the researchers and proved less important than other parameters in fostering the participants’ willingness to participate and share their views. Neither did participants demand the researcher to reveal their religious affiliation (although in some cases this could be assumed based on attire), nor did the researchers make it a point to reveal their identity. For example, one of the researchers self-identified as an atheist. What seemed to matter more was the researchers’ understanding of the community, having pre-existing links to participants and being culturally and politically aware.

Other background characteristics, including social and educational differences, also shaped relationships. For instance, EGK was an educated urban woman from the capital of Ethiopia who had no first-hand experience of irregular migration to the UK or DVA. These differentials could have deterred some of the women from sharing their stories, although this was counterbalanced by the trust that already existed between EGK and the group, which she coordinated. Similarly, being a member of the British Bangladeshi community allowed AA to build a rapport with her study participants. Both AA and her Bangladeshi participants were mostly educated professionals. Nonetheless, recruiting professionals from the local authority turned out to be difficult because they were mindful of not (mis) representing the organizations they worked for, despite being asked about their individual views. Some participants in the Bangladeshi study reported that they often found themselves wanting to help women from their own community access services but being limited by professional boundaries as they often had other roles in the community. For instance, one of the researchers was working in statutory services and it became clear that participants sometimes hesitated to share openly, unsure which “hat” she wore. Her professional role may have restricted them from voicing their dissatisfaction with DVA services. As a general approach, each researcher judged each interview context and probed where possible without compromising their place in the community.

In the Eritrean context too, NP’s links to the community made the research possible. A complex political history, a high level of mistrust among Eritreans in the diaspora due to this history and the divisions it has fostered along political lines, and the sensitivity of the research topic would have made it difficult for a complete “outsider” to conduct such research. Whilst NP was limited by her non-Eritrean identity, an “insider” researcher of Eritrean origin would face perhaps greater challenges because of deeply entrenched political divisions in the community. In this research setting, political positions were often discussed openly, and it helped that NP did not have political grievances or alliances in common with her interlocutors except for a firm belief in human rights and social justice, reflected in her support for Eritrean refugees.

Lastly, all the researchers were female, with most participants being female. The researchers’ gender identity was important for building the necessary trust with female participants, without hindering the inclusion of males in the study. However, it is unclear whether employing a male researcher might have enhanced engagement with male participants.

### Participant recruitment

In total, NP conducted eight interviews with members of the Eritrean communities; AA conducted eight interviews with Bangladeshi participants, and EGK facilitated one FGD that took place over two different days involving six Ethiopian and two Eritrean women. Research participants included men and women in the lay population (*n* = 6), religious leaders serving these communities (*n* = 2), and community members involved in initiatives supporting DVA victims/survivors in culturally sensitive ways (*n* = 16).

To recruit professionals of Bangladeshi heritage working with women and children exposed to DVA, AA sought the support of specialist DVA organizations in London. EGK is the founder of the *Ihit*-*le*-*Ihitoch Habesha* Women’s Support Group (Sister-to-Sister Abyssinian Women’s Support Group) and was personally connected to the participants, whom she recruited. All the women involved in EGK’s study were survivors of DVA and other forms of violence against women and girls, although they had not been recruited with this criterion in mind and were not asked directly about those experiences. Similarly to AA and EGK, NP relied on personal networks for recruiting participants for the Eritrean study. Interviewees hailed both from former professional contexts and personal relationships. Only three participants (two women and one man) were new to the researcher and were recruited through other participants.

Although recruiting through personal networks carries practical limitations and raises ethical considerations, including the risk of perceived or actual coerced participation, confidentiality concerns and potential sampling bias, this approach was considered appropriate as it fostered trust and encouraged openness among participants to share their perspectives and experiences. These qualities were essential in a qualitative study of this nature, where gaining deeper insights into participants’ perceptions depended on rapport. The researchers took steps to mitigate the potential risks by emphasizing the voluntary nature of participation throughout, ensuring confidentiality by creating safe spaces, flexible scheduling, and the option to withdraw at any stage. The research sought to be considerate of the participants’ busy schedules and conflicting priorities, so all interviews and the FGD were conducted online. Formal ethical approvals for the research were granted by SOAS University of London. In recruiting participants, the researchers presented them with a consent form and discussed it thoroughly before obtaining written consent.

### Definitions of key concepts

In line with our community-led research approach and our commitment to prioritize the participants’ understandings, we did not provide a rigid definition of “domestic violence” but rather explored how participants understood the problem. The community-based researchers encouraged participants to speak freely and openly around these concepts and explored definitions together. These encompassed discussions on types of violence and abuse that participants considered most relevant and situations they included or excluded in their definitions. The researchers were open to exploring all forms of DVA that the participants considered important, including violence inflicted by romantic partners and by extended family members.

Similarly, we did not provide participants with a definition of “religion,” a concept that originates in 19th century European thought and whose definition and theorization has been shaped by western European colonialism and secularization processes, hence carrying well-documented biases and assumptions (Deneulin and Rakodi, 2011; Istratii, 2020). We aimed to avoid rigid definitions found in relevant literatures such as religious studies, international development, public health, and social care, particularly those that historically implied a separation between private and public domains, or between the social and the sacred, or assumed specific relationships between “religion” and “culture,” which are context-dependent and variable. We rather explored how participants thought about the influence of religious tradition, religious mediators and personal faith in DVA experiences, making it clear what precisely we were asking each time and probing participants to explain further if they referred to “religion” generically.

In considering matters of definition, it is important also to note language use and translation. NP and AA conducted their interviews in English, which was preferred by participants, whereas EGK carried out the focus group discussion in Amharic, which was her and most participants first language. Participants who spoke Tigrigna were aided with translation by other bilingual participants in the same group. EGK, as the only researcher working in a language other than English, grappled with issues of translation, especially around culturally specific concepts lacking exact English equivalents. For instance, she reported that the term “domestic abuse” (as opposed to “violence against women and girls”) was difficult to translate into Amharic. Using available terms to translate it could unwittingly reinforce a focus on physical forms of abuse. To mitigate this, EGK and the other researchers encouraged participants to reflect on their definitions and consider forms of abuse not commonly discussed in the community.

In presenting the results, we have tried to use the general term DVA consistently throughout the paper, although the participants used several terms, including *domestic violence*, *domestic abuse*, and *violence against women and girls* (VAWG). When presenting direct testimonials, we retained participants’ original terminology.

### Analysis

The transcripts were coded by each researcher under the guidance of RI according to a common list of thematic codes agreed collectively. We used our own coding approach developed through experience working in large teams, combining deductive and inductive logics and processes. A set of primary codes was generated by the lead researcher working deductively from the research questions. This list was communicated to the researchers for further brainstorming, which resulted in its refining. This phase also helped clarify the research questions and aims for all the researchers.

The deductively generated list of codes was then revisited after all the researchers had completed one or two interviews or first FGD meeting to consider necessary alterations. Lastly, the list was finalized inductively once all interviews were completed. This resulted in new thematic codes being identified and some pre-existing ones merged to ensure a cohesive presentation of the results and minimize duplication. The community-based researchers continuously revisited, discussed, and refined the list during data coding. We worked from a live document accessible to all researchers, allowing the team to review, accept, or reject new codes proposed by others depending on overlap or contextual relevance. Most codes identified deductively were maintained, with about a quarter developed inductively.

To synthesize the results, recognizing the time and commitment constraints of the community researchers, each was asked to produce a report presenting their findings under the agreed thematic codes. The community-based researchers were provided with general guidelines of what the final reports should include to ensure that these could be subsequently synthesized with some consistency and a degree of alignment. The team then identified both common threads and context-specific insights. Numerous meetings were held to conceptualize and refine the contents of the final synthesis paper which integrated the three reports into a single publication. This was a reiterative process that involved extensive back-and-forth communication between the lead investigator and the community-based researchers.

A primary synthesis of the findings was compiled by NP with input from the other two community-based researchers, as NP was hired for the project as a long-term researcher and had the capacity to engage in authoring the paper, unlike the other two researchers. In many cases, NP revisited the transcripts to clarify context and meaning. RI drafted the paper and worked collaboratively with NP to present the findings and contextualize them in the existing literature. The paper was then reviewed by the community-based researchers and co-investigators (AA, EGK, PA, and GF). Throughout the data analysis and synthesis process, the team worked collaboratively online to ensure involvement at every stage of analysis, synthesis, and write-up. However, the two community-based researchers could dedicate only limited time after completing their fieldwork due to other professional commitments.

## Results

Participants were diverse in terms of their age, years living in the UK, professions and religious affiliations. The average age for the sample was 40. Approximately one-third of participants had been in the UK since early childhood, while almost another third had spent several decades there. Of the 24 participants, 15 identified as Muslim, six as Christian Orthodox, two as Jehovah’s Witness, and one as not religious (Table 1).

**Table 1.** Participant demographic characteristics.

Participant number	Sex^a^	Age	Country of birth	Years in the UK	Profession	Religious affiliation
P1	Female	43	Eritrea	22 years	NGO worker	None
P2	Male	31	Eritrea	8 years	NGO worker	Muslim
P3	Male	45	Eritrea	25 years	Religious leader	Muslim
P4	Female	49	Eritrea	25 years	Health worker	Muslim
P5	Female	61	Eritrea	30 years	Health worker	Muslim
P6	Female	47	Eritrea	30 years	NGO worker	Orthodox Christian
P7	Female	53	Eritrea	32 years	Social care worker	Muslim
P8	Female	58	Eritrea	35 years	Community worker	Muslim
P9	Female	51	Bangladesh	From early childhood	Religious leader	Muslim
P10	Female	55	Bangladesh	From early childhood	Local authority	Muslim
P11	Male	Not declared	Bangladesh	From early childhood	Local authority	Muslim
P12	Female	49	Bangladesh	From early childhood	Local authority	Muslim
P13	Female	28	Bangladesh	From early childhood	DV organization	Muslim
P14	Female	31	Bangladesh	From early childhood	Local authority	Muslim
P15	Female	45	Bangladesh	From early childhood	DV organization	Muslim
P16	Female	32	Bangladesh	6 years	DV organization	Muslim
P17	Female	27	Ethiopia	4	Support worker	Jehovah’s Witness
P18	Female	23	Ethiopia	6	Full time mother	Orthodox Christian
P19	Female	29	Ethiopia	8	Full time mother	Orthodox Christian
P20	Female	24	Ethiopia	5	Full time mother	Orthodox Christian
P21	Female	35	Ethiopia	4.5	Full time mother	Muslim
P22	Female	23	Ethiopia	3	Full time mother	Orthodox Christian
P23	Female	24	Eritrea	7	Full time mother	Orthodox Christian
P24	Female	63	Eritrea	8	Support worker	Jehovah’s Witness

^a^Participants were not asked to self-identify their gender, hence only sex is mentioned.

### Beyond physical violence: Participants’ understandings of DVA

In all the study contexts, understandings of DVA encompassed more than physical violence, although visible physical abuse was often the most readily recognized form. For many participants, DVA also included psychological, sexual, and financial abuse, as well as mistreatment by other family members, particularly mothers-in-law. Participants’ awareness of DVA was influenced by numerous factors, including exposure to women’s rights language, relevant legislation in the UK, and socially prescribed gender norms cut across the Bangladeshi, Eritrean, and Ethiopian communities. Many participants explained that their understanding has evolved over time because of their stay in the UK.

In the Bangladeshi community, there was variation in the language used to describe DVA, but also a notion that abuse was taking place only when there was evidence of physical harm, as highlighted in the account below:

Majority of the time people are focused on (physical abuse), and it is easier for them to deal with it if a woman has a bruise. It’s easier to say, ok you are a victim of domestic abuse. If she is experiencing sexual abuse for example, would she go to an imam and say, look my husband is sexually abusing me. No, she wouldn’t. (Bangladeshi study participant)

The general perception among the Bangladeshi participants was that other forms of DVA that were not visible, including sexual abuse, tended to be understated in the community.

Among Eritrean participants, many said they had to educate themselves about DVA after arriving in the UK, whilst a few female and male interlocutors said that DVA was not a problem as they had not met women subjected to physical violence. Even so, perceptions in this context too often initially focused on visible physical harm.

Similar ideas were expressed within the Ethiopian study context, as illustrated in the following account: “I have learned in this country, some women don’t even know [that] they are being abused.”

Within the Eritrean and Ethiopian community contexts, participants explained that traditional gender roles ascribed to women were constructed around perseverance or endurance, with many proverbs and sayings promoting women’s submission and justifying a “machismo” type of masculinity, as some research participants put it. In some cases, such attitudes were reinforced by the discourses and responses of religious figures, such as spiritual fathers, as illustrated in the following account: “When they tell their family members [about abuse], they are told that they should be patient and endure it as it’s just the way it should be. Some go to their priests seeking for advice and they will get advice to endure the situation.” (Ethiopian study participant).

A Bangladeshi research participant also described women’s submission to men as a community-wide norm:

[I]t’s so culturally, you know, ingrained that it’s the norm, normal behavior to treat women in a degrading manner, in a manner where she has to perform some duties (physical and sexual), and it’s expected of her. Just there now, it’s not seen as a misuse of cultural or religious norms. It’s just natural, and it’s expected, and she expects it too, as well as her family and he (husband) and her family; it seems to be like from my experience that it’s like you would do, it’s unusual if it doesn’t happen.

Participants explained that often adults had witnessed abuse as children, and this contributed to its normalization within families and communities. Since their parents had perpetuated such behaviors and norms, asking for their support was believed to be futile. One participant said:

[…] if there is any kind of instances where perhaps a woman in that situation goes back to, say in a most likely to say to her mum and says, you know this is what’s happening in my relationship with my husband, if the mum has been subjected to those kind[s] of treatment the likelihood of mum then just saying, it’s nothing unusual, you’ve seen how your dad treats me and so that’s how men are, so just accept it, just bear it, and that’s the thing some women accept. (Bangladeshi study participant)

Ethiopian participants also reported that certain forms of verbal and psychological abuse, such as intimidation, belittling, and financial control were normalized. Some participants described men as victims-turned-victimizers, who had been socialized in an abusive environment and had unhealed trauma, as the account below illustrates:

It’s because of the culture—I don’t think they are aware that they are being violent. I think it’s because of their upbringing and the trauma experienced before they came to the UK or what they have been through when they migrate to the UK. The men themselves need a lot of therapeutic support. When they grow up, they might have experienced domestic abuse within their family or in their neighborhood and they think it’s how you should treat women. (Ethiopian study participant)

Participants also explained that many Ethiopian and Eritrean men arrived in the UK via treacherous migration routes, crossing into Europe through the Sahara Desert, Libya, and the Mediterranean Sea, and may have been subjected to human rights violations. They believed that such abuses led to unhealed trauma that manifested as violent behavior, as the following account illustrates:

It could also be the psychological trauma that affect[s] them during the journey. We hear a lot of horrific stories [about] what happens in Libya: both men and women are raped, their kidney forcefully stolen and all sorts of stories. [...] I think they displace their anger and frustrations on their wives and girlfriends. Some people take out their anger by kicking their dogs, or on the door, I think that’s why they are like that. Because they have a lot of anger, sadness and misery bottled up inside. (Ethiopian study participant)

While participants attributed violent behavior to trauma experienced during precarious migratory journeys, they also conveyed an understanding that abuse and violence tend to arise from complex, intertwined origins encompassing pre-existing cultural, familial, and social patterns preceding the migration experiences.

Eritrean participants agreed that financial problems were a major cause of DVA. For example, a woman might send money back home, and her husband might object, leading to disagreement and even physical violence. Additionally, while both female and male refugees are entitled to asylum and welfare benefits, participants reported cases where men exercised control over the family income. Other study participants explained that family reunions and arranged marriages between UK-based Eritrean men and Eritrean women wishing to escape from refugee camps and urban displacement in East Africa could also result in power imbalances between spouses, as well as unmet expectations among women arriving to an unfamiliar environment. An example is provided below:

So, the man had settled and adjusted and knows about the system. The woman comes as a guest. She’s under the control of the man. So that’s more hurdles for the woman, the man would say “I brought you here and now you’re using me” type of thing but also knowing how to go and get help. You know that they go by years and years without knowing anything, because they’re grateful that the man had brought them here and you know, he’s just the Almighty basically. He didn’t bring them just to benefit the woman, but to benefit him as well. Even in the community, you get that extra respect if you bring your wife here. So, for her to go on and accuse him of abuse, wow, no way. (Eritrean study participant)

Overall, in all study contexts participants expressed a nuanced understanding of DVA that extended beyond physical violence to include psychological, sexual, and financial abuse, as well as coercion and mistreatment within wider family networks. Their accounts highlighted that DVA arises from interrelated factors including systemic inequalities, harmful gender norms, intergenerational patterns and migration-related experiences affecting both women and men. While gendered power imbalances were central, men were generally seen as being under-served by the same normative, often patriarchal and highly stressful, traumatic system, that trapped them in the embodiment and perpetuation of toxic and even violent masculine behaviors.

### Multiple perpetrators within the extended family

In most accounts, participants identified multiple perpetrators within extended family settings. These dynamics were reported as operating both within UK-based households and communities, and also through transnational family ties.

This was particularly prevalent in multigenerational families living in the UK and emerged as a recurrent issue in the Bangladeshi community. In this study group, participants spoke about a hierarchical relationship between wives and other women in the family, with elders, and especially mothers-in-law, occupying a more powerful position. Daughters-in-law were often subjected to controlling behavior by mothers-in-law, which could contribute to multilayered oppression within the larger family, as the account below shows: “So, one of the things I’ve seen there is not only domestic violence in all its forms, from the spouse, but it can happen from the extended family members, like brother-in-law, sister-in-law’s, mother/ father-in-law’s.” (Bangladeshi study participant).

Similar issues were raised in the Ethiopian context, where mothers-in-law were described as having direct influence on marriage dynamics, which could, in turn, lead to wives experiencing emotional pressure or abuse by their husbands. Husbands would often compare wives to their own mothers, praising their mothers for having been the “perfect women” despite hardships that they had experienced back in Ethiopia. Those were often juxtaposed to their wives’ improved conditions in the UK. One participant described the following incident:

I used to have severe morning sickness when I had my first baby and he used to tell me that I should stop complaining because his mother had a tough life and was much stronger: “but you don’t have a busy day, you spend most of your time sleeping. Why are you complaining? […] You don’t do a quarter of what she is been through.” (Ethiopian study participant)

Such comparisons often contributed to fostering wives’ antagonism or even resentment toward mothers-in-law and caused arguments between spouses. Comparisons could also be made between sons and fathers by mothers-in-law to silence women into submission, as in the case below:

For example, I bought [an] Ethiopian traditional dress and wanted us all to have a professional picture. But he kept postponing it because as you know, he doesn’t like spending money and how he controlled all the expenses. But she (husband’s mother) said to me that “you can live with him peacefully; he is much better compared with his dad.” Usually, couples compare themselves with other peer couples, but my competition was with his parents. It felt impossible. (Ethiopian study participant)

Participants’ accounts underscore the significant role of hierarchical structures among females within the same household, with mothers-in-law exerting substantial authority over daughters-in-law, influencing marital relationships and even contributing to experiences of abuse. This hierarchy not only perpetuated multilayered and intergenerational oppression, with mothers-in-law reproducing the abuses they had experienced in a rigid and unequal patriarchal system in their interaction with younger females, but was also seen to complicate women’s help-seeking, as familial control often discouraged disclosure and encouraged endurance, most markedly in the Bangladeshi and Ethiopian contexts.

Simultaneously, participants emphasized that pressures were not confined to the household or local community but extended through transnational networks. Even in cases where relatives were not physically present in the UK, they could still have an impact on the married couple. This was particularly evident in the Eritrean community, which participants presented as a closely knit across cities in the UK and beyond, as illustrated below:

The news travels back home, so their families back home would be another barrier [to reporting a perpetrator]. So, if a woman does anything, the man will definitely tell his family and her family, and then it would be, why are you reporting your husband to the authorities, so there’s a community issue, and a culture issue here. It’s local, it’s national, people in the UK know each other, the community is very interconnected. Whatever happens in London will be known to someone who lives in Birmingham, Sheffield, or Leeds. So, you have this blame and shame aspect as well. (Eritrean study participant)

Since news tends to travel “back home” quickly, pressure can be exerted on women by relatives overseas to act in a socially ascribed manner. Such transnational pressures were reported to reinforce ideas that women were to blame for marital problems, that they were responsible for “fixing” them, and stigmatized those who left abusive partners.

Participants believed that this complex nexus of power relationships, spanning households, diasporic communities, and transnational family ties, was rarely considered or understood by DVA service providers. As a result, this limited the latter’s ability to effectively support women facing multiple perpetrators or webs of oppression within the extended family.

### Division between the private and the public sphere and notions of shame and honor

In all study contexts, participants articulated that DVA was treated as a private family matter. Across these communities, it was widely believed that a woman who spoke about DVA failed to fulfill her social role as a “good” wife, mother, or woman. The following account is one of many that were reported to the researchers:

[I]f a woman is a victim, […] she would speak to somebody older who could maybe talk to her husband or something like that. But they definitely wouldn’t want these to come out and go to a sort of organization or if there is any kind of help organized, like domestic violence helpline or something, they would not be alerting them to. They will still consider that this is a private matter. (Bangladeshi study participant)

Furthermore, participants explained that women who did speak out risked further abuse in the extended family or community context. In most cases, a conversation about DVA would be considered an intrusion into a family’s private life and women would have no option but to remain silent. The participants’ accounts suggested that people who were aware of DVA cases would be hesitant to intervene, in some cases were explicitly asked by the victim not to do so. In the Eritrean community, for example, participants presented a picture whereby everyone exerted effort to keep family issues private. As a result of gender biases that were described as widespread, when things went wrong people narrowed down on women’s and not on men’s actions, with women being seen as disobedient and as bringing shame. A participant explained:

And culturally we think that family… that no one should speak about the family, you know. It’s forbidden, it’s [a] shame to speak about what happens in the family, whether the couple, or the family unit, you don’t talk about it outside home. So, this being secretive… which is again, common in many communities, not to single out any community, whether it’s Muslim or Christian. (Eritrean study participant)

An Ethiopian participant also explained that taking formal action against a perpetrator was considered a taboo and that she herself had faced social ostracism when she decided to leave her abusive husband. Another Ethiopian participant recounted that those experiencing DVA would be criticized in their Orthodox Church congregation if they pursued legal avenues to deal with perpetrators. One woman affected by DVA reported that she had been advised to manage the problem amicably, and to forgive her abusive partner as a good Christian should do.

Expectations about prioritizing family values (as opposed to individual needs or desires), and notions of shame and honor continued to be predicated on women’s behavior. According to the study participants, upholding the reputation of the family was the sole responsibility of a dignified woman. In the Bangladeshi community, participants also reported that if DVA occurred, it was assumed that there was something wrong with the woman and if she spoke out, she was believed to behave inappropriately:

And there’s such a stigma, you know, around it, you know as I said before, I think it’s such an expected thing, you know, it’s just it’s the norm, and if you kind of speak out its kind of like there’s something attached, it becomes something that is attached to the woman where she is kind of misbehaving, or there must be something wrong with her (Bangladeshi study participant).

In some cases, the expectation for women to endure DVA situations was coupled with a belief that Allah would reward their patience and endurance. Participants’ accounts also suggested the existence of a wider belief that not talking about the abuse would eventually result in the problem disappearing.

In the case where DVA victims mustered the courage to seek help within religious establishments, they would often be advised to work on their marriage despite the abuse continuing. Research participants in the Ethiopian and Eritrean cluster explained that whilst both Christian and Muslim religious leaders and elders in their communities often reprimanded a violent husband, the onus was still placed on the wife to endure her situation. Participants reported that abusive men did not follow through with the agreed corrective actions, evidencing the limited power of religious mediation. The account below is illustrative:

Especially if his family has a status in the community, there is no point going to the faith leaders. They tell him off, counsel him but men like that have narcissist behavior so he pretends as if he is good. He says “I haven’t done anything wrong, it’s her who did something wrong; it won’t happen again” but the abuse didn’t stop all her life. But my mother ran away to the city leaving him behind. That’s when the abuse stopped. (Ethiopian study participant)

And in spite of what were often lengthy mediation processes, abusive situations could escalate, putting the life of a woman and her children at risk. These processes were described as prolonged because women were repeatedly encouraged to forgive, reconcile and remain in the relationship, even when violence persisted. Mediation appeared to focus less on resolving abuse and more on maintaining family unity and preserving the family’s reputation. Participants’ accounts suggested that this emphasis on endurance reflected broader cultural pressures on women to embody patience and sacrifice, often framed in religious terms, such as being a “good wife” of “good Christian/Muslim,” which meant that women were left with few acceptable alternatives except staying in the relationship. One Eritrean participant explained:

[…] after years of abuse and years of mediation by family members, local community leaders or religious leaders, priests, or sheikhs, they fail to resolve the issues and then it goes out of hand. […] The priests and sheikhs try to solve the problem by keeping the family together, but then children get involved, the man who is abusive towards his wife is in the majority of cases also abusive towards his children, and children will go to school with bruises, and things escalate from there, for example. (Eritrean study participant)

Overall, participants presented a picture of a community-wide tolerance of DVA, which traditional mediation processes did not eschew. These deeply ingrained gender expectations were described as different from the UK’s statutory environment and gender equality standards. One of the Eritrean participants recounted a story of a young Eritrean woman who came to the UK to marry an older man who turned out to be violent. Her story illustrates tensions between gender standards at home and the UK’s statutory environment, especially in relation to safeguarding children:

She is very young, and she came with a child from back home, a child from another relationship. This man was violent towards this child. […] And I happened to know this man in the community. He was one of the violent men, known in the community. So, I went to him and said, in this country it’s like this. And I said to this woman, this man is hot tempered, how do you manage his temper? And she told me, when he becomes fire, I become water. And I said, if you put too much fire in the water, the water also boils. […] And a few months later she had a nervous breakdown, she was admitted to hospital, the children were taken away to foster care. […] So, the water can also boil. I will never forget it. This is what the society expects from women culturally.

Interactions with the UK statutory and cultural environment clearly impacted community-upheld gender norms, which merits further research beyond the current study.

### Male-dominated religious establishments as a barrier for women seeking help

When study participants were asked to share their thoughts on the response of religious institutions to DVA, they tended to present these as male-dominated and as less accessible to women. Since such institutions were comprised or led by men, women hesitated to approach them, as the two accounts below suggest:

So, he is an imam and he is a man and in some respect this is a big barrier for them (women), doesn’t help the fact that most of their community leaders are men, which is quite disproportionate, actually because then you know, the issues come into that, talking to a man about abuse. (Bangladeshi study participant)

Some women who come to report DV are scared of the men themselves. They try to avoid men. (Eritrean study participant)

Speaking about the traditional Eritrean Orthodox community context, a research participant felt that there was a general sense that the members of the congregation and the priests had a mostly public relationship, and that encounters between the two happened only on special occasions. They explained:

The relationship with a priest is quite formal, very rigid. Unless you have a christening or wedding to plan, you wouldn’t approach a priest. If you do, it’s like, oh I spoke to a priest today, it’s a big deal kind of thing. You don’t have an interaction with [a] priest apart from that. […] It’s like, it’s almost like they’re closer to God. So, you don’t approach them unless you have a very good reason. And domestic violence is not considered a good reason. (Eritrean study participant)

Similarly, approaching a religious institution would not be easy for a Bangladeshi Muslim woman. Mosques were described as male-dominated spaces where women’s voices were not listened to. Similar issues were raised by Ethiopian participants in situations where abusive husbands happened to be serving in the Church as priests. Consequently, many women sought solace personally through their faith, prayer and spiritual life rather than within their respective institutional religious settings. In the Eritrean and Ethiopian communities, and to a certain extent also in the Bangladeshi community, community elders were also reported to have considerable influence on the family life. Those structures were described as more informal, but they were perceived to be equally male dominated. The two accounts below illustrate the issues:

Of course, I don’t expect anything else, most mediators are men, so it’s not surprising that they can’t resolve those issues. Most, if not all community leaders and religious leaders are men. And they fail to resolve a problem and things escalate. Or women give up and decide to live like they used to. (Eritrean study participant)

When you approach elders/priests or the sheikh (imam), even if she was physically abused no one encourages you to go to the law. Even if the woman wants to seek help, they discourage her and silence her. The mediation usually ends up with the woman to be patient and forgiving and he would never do it again. Every time it happens, they tell him he will get better. It’s a taboo to tell her to go to court. (Ethiopian study participant)

While not all religious mediators and clerics responded in the same way, the perception that they also adhered to the same cultural standards as others in the community was strong in all study contexts.

### Keeping the family together and marriage as an institution ordained by god

Cultural expectations fixated on women’s endurance when dealing with an abusive intimate partner made it harder to challenge perpetrators. Participants’ accounts suggested that women’s silence could be reinforced in reference to faith-based conceptualizations of marriage as ordained by God where the community deployed these understandings to limit women’s options. The accounts below are illustrative:

[I]n [...] our culture where faith is a big thing where, you know marital relationships are conducted under faith-based oath. Yeah, so that’s a sacred matrimonial relationship, which requires spiritual and religious leaders to advocate a break or authorize a divorce, professional and also the faith-based element that requires before even get[ting] to that stage of you know, separation, right? (Bangladeshi study participant)

Because marriage is ordained by God so it’s very very hard psychologically, mentally to contemplate breaking that. (Eritrean study participant)

Many Eritrean participants felt that religious teachers and mediators, either Muslim or Christian, were reluctant to advise divorce since this is not a formal religious teaching and because the clergy prioritized the continuation of marriage, as the following account suggests:

I would say religious leaders play a big role. Sometimes they would suggest a divorce when there’s no point in pursuing a relationship, but most of the time they are driven by the fact that they want to preserve the family. So, it might be a very subtle factor that affects the mediation process. So, they are doing their best to come to terms with the situation, to make some arrangements so that a relationship could be saved. (Eritrean study participant)

In all research contexts, divorce was perceived to be sinful and socially unacceptable. Therefore, if a woman reached out to her priest or imam for help, it is likely that she would be advised to work out a solution together with her husband, although several participants recognized that clerics were there to support the preservation of marriage. An Ethiopian participant, for example, explained:

In our faith (Ethiopian Orthodox), I don’t expect the priests to encourage divorce or separation because marriage is holy and respectful. It is very difficult for them to advise separation; they might have to support them, but I don’t expect them to tell the couple to get divorced or separate. If I go to the church seeking help, I expect them to give us spiritual advice and discipline to help the relationship get better, not to tell us about separation.

It should be stressed that these were the experiences of our research participants and should not be generalized. Other studies from the Ethiopian rural context, for example, have found that priests may not interfere with divorces, although they will not advise them directly (Istratii, 2020). It could be that in the diaspora context, priests’ responses may be more orientated toward preserving marriage as the primary unit of the community, although this would require further research. It may also be considered that in the UK, the role of religious leaders is not typically a full-time job, which would restrict their involvement with the community.

Regarding help-seeking barriers, Ethiopian study participants also proposed that women did not want to leave an abusive relationship because they feared that their children would grow up without a father. A participant recounted a story from her congregation about a woman who refused to leave her husband because she did not want her children to blame her for family breakdown. The pressure to keep the family together because of children was also present in the Bangladeshi community, as illustrated below:

So, they would much rather be making the marriage work for the sake of the children is what you always hear. I’ve got to stay in this relationship, I don’t want to be the single parent, I don’t want people to know I’ve got my husband out, so those kind of elements and social pressures and shame comes into effect when people are kind of trying to deal with issues.

Bangladeshi study participants also generally believed that women’s rights in Islam were often mispresented by the religious leaders. Their accounts suggested that religious leaders could contribute to the culturally accepted norms about men’s violence against women, maintaining male domination. The study suggested that women’s voices were seldom present within religious injunctions related to marriage, divorce and leaving a violent relationship. Similar to the Eritrean and Ethiopian communities, Bangladeshi study participants agreed that the focus of religious leaders had been on keeping the family together.

### Perceptions about services not fully addressing the needs of women from diverse migrant communities

Participants’ accounts suggested a perceived lack of accessible support services for women. The women who participated in the Ethiopian focus group were not aware of any such services. Although they reported the existence of an Ethiopian community center based in North London and a small charity called Women Empowerment Group in West London, and some informal initiative by women in the Ethiopian Orthodox church, they did not know of any dedicated domestic violence service targeted to these communities.

According to Bangladeshi study participants, the service provision landscape for their community in the UK has also been patchy, particularly when tackling DVA incidents and homicides that often remained hidden. Racial discrimination, Islamophobia, and a lack of cultural understanding were mentioned as barriers in accessing mainstream services. The study participants reported that staff in statutory services struggled to understand Bangladeshi women’s reluctance to report abuse as perpetrators’ power of retribution generated fears for the victims.

Additionally, research participants from all study clusters believed that their compatriots did not trust social services, which were commonly regarded as “taking children away” and “destroying families.” To minimize this risk, women who experienced abuse but did not seek direct DVA support, instead mentioning children’s problems at school or health issues when communicating with health practitioners.

The accounts of Eritrean participants suggested that the welfare and asylum systems in the UK could also reinforce power imbalances between men and women due to social housing and welfare benefits being under the name of the husband. Even when a couple arrived together to the UK and claimed asylum together, they would not typically share equal rights as the norm would be for the husband to appear as the main applicant:

People come here and they don’t know the system, they are used to a specific way of life. In that system, a man is a breadwinner, and a woman looks after their family, there is no other income. But here you have a completely different system. There is a possibility of a woman working as well, and that’s where the shift is happening. Also, if it is a family reunion, a man arrives here and then brings his wife, she wouldn’t necessarily have access to public funds. She would have no recourse to public funds and technically, she’s dependent on a man. And that gives a man full control and power. And that makes a relationship very toxic, in terms of power imbalance (Eritrean study participant).

Ethiopian and Eritrean study participants additionally explained that there were women in their communities that had been married only in church whose relationship status was not recognized as legal in the UK. There was also a fear of deportations among newly arrived women being manipulated by their abusive partners who had been in the UK longer.

### Gaps in, and need for, community responses

In considering improved responses to DVA, participants agreed that there was a need for speaking openly about the problem and improving awareness of VAWG and DVA laws among both women and men. They also spoke about the importance of working through religious leaders and clerics, as illustrated in the accounts below:

Yeah, I think, you know, definitely, we need to really be talking about it [domestic abuse] more. We need to talk about this as an issue. This should not be one of many things which are just either brushed under the carpet or, you know, seen still as a taboo. (Bangladeshi study participant)

I think it’s always good to raise awareness, as I said. Violence against women, the root cause of it is power imbalance, patriarchy and all that. They need to understand that. It’s only through educating that we can address those issues. One, we educate those religious leaders and the whole community. That might help to reduce incidents of violence. (Eritrean study participant)

When people first come to the UK, they get orientations. Maybe they should support religious leaders to go and teach them about this. Education should be given not only on faith-related issues but other practical areas too. They may not change everybody’s heart, but they can influence many of them. (Ethiopian study participant)

Many believed that clerics and religious scholars needed specific training to understand the extent and impact of DVA on families and to address it through their sermons and regular teaching.

For example, participants suggested that each Ethiopian Orthodox Church’s *sebeka gubae*, an office that support administrative church work, could train two or three members to respond to DVA and refer women to support services such as shelters or refuges. Participants also viewed mosques as well placed to offer Islamic guidance and advocate for women’s rights. However, they observed that imams rarely discussed the shared responsibilities of men and women within the family or their equal contribution to family harmony. Most imams served as spiritual mediators without formal training or institutional support. Participants believed that improving imams’ knowledge of Islamic teachings could strengthen faith-based efforts to prevent VAWG.

Participants believed that faith leaders should consider more seriously all forms of domestic abuse, not only physical harm, recognize the risks to women’s and children’s lives and wellbeing, and act promptly where needed, instead of encouraging women to deal with the issue as a private matter. As an example of good practice, a family committee was reported to have existed at the Eritrean mosque in London. The imam who was interviewed for the study was trained in family therapy and held a degree in counselling. The mosque he belonged to encouraged members to come forward if they needed help and took initiatives internally, such as by discussing marriage problems during Friday sermons and holding lectures and seminars on resolving family conflicts.

These accounts suggest that engaging with clerics and religious leaders through education and community facilitation could be crucial. Participants emphasized that the ways in which faith is personally interpreted and practiced shapes religious leaders’ responses to DVA, either facilitating or hindering productive responses to DVA. The variability in how religious leaders and clerics interpret and enact the religious tradition they uphold can significantly affect the quality and effectiveness of DVA responses at community level. Recognizing and navigating appropriately these variations by means of theologically grounded clergy training approaches could enhance their responsiveness of faith-based approaches to abuse.

## Discussion

The findings showed that understandings of DVA were disproportionately focused on physical violence. However, participants’ understandings broadened over time due to their stay in the UK and exposure to women’s rights language and relevant legislation. They increasingly came to understand DVA as encompassing psychological, sexual, and financial abuse, as well as mistreatment by extended family members, particularly mothers-in-law.

Despite the diverse religio-cultural backgrounds of the Ethiopian, Eritrean, and Bangladeshi participants, the findings showed a cross-cultural tendency among victims and those around them to be generally secretive and private about DVA. They all pointed to the role of socially ascribed gender norms in deterring victims from taking formal action against perpetrators or from exiting abusive situations. This was compounded by the normalization of abuse within families and communities, often because adults had witnessed abuse as children. Normalization was reinforced by cultural expectations of women’s perseverance and submission, justifying a “machismo” type of masculinity, as some participants put it. Participants also attributed some violent behavior to unhealed trauma experienced by men during treacherous migration routes, as well as to financial problems, power imbalances in family reunions and arranged marriages and the UK’s welfare system, which often assumed men to be in control of household income.

Legal and economic precarities, limited knowledge of UK laws, and patriarchal family norms were believed to contribute to women’s dependence and vulnerability to DVA. We found that victims/survivors must navigate deeply rooted society-of-origin social norms and religious expectations around marriage, the conjugal relationship, and the family dynamics, which continue to have a stronghold in the migration context and to circumscribe women’s options. Notably, participants also reflected that men themselves were underserved by these patriarchal and traumatic systems, which perpetuated toxic masculine behaviors while simultaneously preventing them from seeking support.

These findings echo existing evidence that migrant and ethnic minority women’s understandings of DVA are shaped by intersecting cultural, religious, and socio-economic factors (Gill, 2004). The participants of this study recognized abuse to include physical, psychological, sexual, and financial violence, as well as mistreatment within extended families, reflecting patterns reported in previous research (Gebreyesus et al., 2018; Khelaifat, 2018; lemma et al., 2023).

Community responses to DVA were limited and often relied on family members, elders, or religious leaders for informal mediation. Such mechanisms were perceived as male-dominated and ineffective, prioritizing family unity and reputation over women’s safety.

Although clerics or elders were perceived as having the ability to reprimand abusive husbands, the burden largely fell on wives to endure the abuse. Abusive partners often failed to follow through on promises of change which was the resolution of most mediation processes, leading to escalation and ongoing risk for women and children.

The findings also deepen our understanding of why members of migrant communities may not make full use of DVA services: they may not feel supported in a manner that addresses their culture-specific circumstances, which they understood as being intertwined with their faith (echoing Holtmann and Rickards, 2018; Istratii et al., 2024). All the participants reported that women affected by DVA were reluctant to report it because they feared that this would bring collective shame. This echoes research by Gill (2004) emphasizing the inhibitive force of cultural norms and reinforces studies that have proposed that overcoming shame as a DVA victim is a prerequisite to acknowledging the guilt of a perpetrator (Saul, 1972; Yamawaki et al., 2012).

These deeply ingrained norms may, in fact, worsen women’s conditions in interaction with a very different DVA response system and statutory environment in the UK. Reported barriers included racial discrimination and a bias toward Islamic communities, distrust of social services due to fears of child removal, a lack of cultural sensitivity among staff, welfare structures reinforcing men’s power and a fear of deportation for women in unrecognized marriages.

Participants overall agreed that extensive awareness-raising campaigns were needed to highlight how DVA endangers women’s lives, harms children’s wellbeing, and undermines community cohesion. They emphasized that effective change would require collaboration between secular and religious stakeholders.

Participants’ accounts also made it evident that religious standards and personal religious beliefs shaped DVA experiences and responses significantly. Participants described how faith could provide solace and resilience for women, but also repeatedly noted that religious institutions tended to be male-dominated and not seen as safe spaces for disclosing DVA. Clerics were perceived to prioritize marriage preservation as an institution ordained by God, advising patience, forgiveness and endurance rather than separation or exiting an abusive situation. Religious mediators were frequently seen to reinforce community norms of endurance, sometimes misrepresenting religious texts, particularly in Islam, discouraging women from leaving. The study highlighted the limited preparedness of most clerics, who lacked awareness of safeguarding, trauma-sensitive counselling, or the theological confidence to challenge entrenched patriarchal interpretations.

However, participants also recognized that faith leaders could play a positive role if properly trained: they could challenge harmful norms, provide trauma-sensitive counselling, and offer referrals to specialist support. Participants often viewed appropriate responses as a combination of spiritual guidance, moral teaching, and practical support, such as mediation or counselling, but these responses were considered effective only when they respected cultural values and offered genuine protection. They proposed that clerics would need to build knowledge about DVA, trauma-sensitive counselling and safeguarding to assist victims and to signpost them to specialist support. Such findings are consistent with recommendations presented in a study by Sullivan et al. (2005: 936) with Ethiopian DVA survivors, in which participants suggested using religious fora to tackle DVA. Bangladeshi research participants, in turn, stressed the need for clerics to teach about women’s rights in Islam, but presented reservations about how such teachings would be conveyed to the community, which echoes Salkic’s (2015: 8) comment on Islamic religious leaders being influential but often ambiguous figures.

## Conclusion

The current study demonstrates the importance of integrating religious parameters in providing culturally resonant DVA services. The findings highlight the limited preparedness of most clerics, regardless of faith tradition, to respond effectively to DVA, and the need to raise greater awareness about DVA within religious institutions and communities. Such work requires broad community engagement to address the gendered and patriarchal foundations of norms surrounding marriage and family. Recognizing the intersections between religious traditions and individual interpretations could help identify effective strategies for improving DVA responses.

Recommendations put forth by Bangladeshi study participants reiterated the importance of engaging DVA professionals from the same community to reverse the existing distrust of statutory organizations. They also emphasized involving female Islamic counsellors and advocated for couple counselling sessions grounded in the Islamic Hadith and Sunnah traditions to address the knowledge gaps around women’s and men’s responsibilities within a marriage and the wider family setting. Eritrean interlocutors, in turn, recommended community workshops framed around “family life,” led by religious leaders who would draw on religious texts to reinforce the message that domestic abuse in all its forms is unacceptable and to stress that women have a right to leave abusive relationships. The study also highlighted good practice examples, such as an Eritrean mosque in London where the imam, trained in family therapy and counselling, openly addressed marriage problems in sermons and facilitated seminars on family conflict, illustrating the potential of informed faith leaders to be effective agents of change.

### Limitations

It should be noted that the research was conducted by three different researchers working directly with different communities, each of whom had different resources. Although they all worked under a common protocol developed collaboratively with the principal investigator, the distinct circumstances of each meant that a degree of misalignment or context-specificity was inevitable. This is an unavoidable limitation in research committed to locally led, community-grounded approaches, which are inherently complex and may diverge from initial research questions as field realities evolve. While the principal investigator worked closely at all stages of the research with the researchers to ensure that they all followed the agreed research protocol and that any adjustments or deviations were communicated and integrated through iterative refinements, the reader may still perceive some lack of full integration or standardization in the research process.

In addition, the research was small-scale and should not be considered representative of the communities included in the study or of the UK’s wider migrant and ethnic minority populations. For example, all the participants were based in London at the time of their interviews/FGD. London has a high representation of ethnic minority communities and features more culturally DVA specific services than other parts of the UK. While participants themselves made numerous generalizing comments about the realities of DVA in their communities, these should not be read as representative of everyone’s opinions or experiences. By engaging deeply with specific communities through culturally and linguistically informed research, the study’s main value lies in providing a window into how religious and cultural context shape access to and engagement with DVA services in different faith communities in the UK at a particular time in history. In authoring the paper, efforts were made to communicate findings with this heterogeneity in mind.

Lastly, this paper has focused primarily on presenting the study in an academically rigorous but accessible manner centered on participants’ lived experiences rather than on extensive theoretical integration. Informed by a decolonial ethos, our aim was to share findings in language accessible to the participating communities, enabling them to benefit directly from the research. Academic scholars are welcome to engage with our findings theoretically or empirically, particularly through alternative epistemological lenses, but our research sought to serve the involved communities first, recognizing that lay communities often conceptualize issues in contextual rather than theoretical terms relevant to their lived realities.

## Note

1. As in our previous paper (Istratii et al., 2024), we use the terms victim/survivor to distinguish between individuals who are in a situation of victimization and those who have managed to exit an abusive situation. We recognize that both victims and survivors can be resilient and actively practice their agency to navigate their respective situations.
